# A Structure Identification and Toxicity Assessment of the Degradation Products of Aflatoxin B_1_ in Peanut Oil under UV Irradiation

**DOI:** 10.3390/toxins8110332

**Published:** 2016-11-12

**Authors:** Jin Mao, Bing He, Liangxiao Zhang, Peiwu Li, Qi Zhang, Xiaoxia Ding, Wen Zhang

**Affiliations:** 1 Oil Crops Research Institute, Chinese Academy of Agricultural Sciences, Wuhan 430062, China; maojin106@whu.edu.cn (J.M.); xinxianghebing@163.com (B.H.); liangxiao_zhang@126.com (L.Z.); dingdin2355@sina.com (X.D.); zhangwen@oilcrops.cn (W.Z.); 2 Laboratory of Quality & Safety Risk Assessment for Oilseed Products (Wuhan), Ministry of Agriculture, Wuhan 430062, China; 3 Key Laboratory of Detection for Mycotoxins, Ministry of Agriculture, Wuhan 430062, China; 4 Quality Inspection & Test Center for Oilseed Products, Ministry of Agriculture, Wuhan 430062, China

**Keywords:** aflatoxin B_1_, photodegradation product, TQEF-MS/MS, cell viability, furan rings

## Abstract

Aflatoxins, a group of extremely hazardous compounds because of their genotoxicity and carcinogenicity to human and animals, are commonly found in many tropical and subtropical regions. Ultraviolet (UV) irradiation is proven to be an effective method to reduce or detoxify aflatoxins. However, the degradation products of aflatoxins under UV irradiation and their safety or toxicity have not been clear in practical production such as edible oil industry. In this study, the degradation products of aflatoxin B_1_ (AFB_1_) in peanut oil were analyzed by Ultra Performance Liquid Chromatograph-Thermo Quadrupole Exactive Focus mass spectrometry/mass spectrometry (UPLC-TQEF-MS/MS). The high-resolution mass spectra reflected that two main products were formed after the modification of a double bond in the terminal furan ring and the fracture of the lactone ring, while the small molecules especially nitrogen-containing compound may have participated in the photochemical reaction. According to the above results, the possible photodegradation pathway of AFB_1_ in peanut oil is proposed. Moreover, the human embryo hepatocytes viability assay indicated that the cell toxicity of degradation products after UV irradiation was much lower than that of AFB_1_, which could be attributed to the breakage of toxicological sites. These findings can provide new information for metabolic pathways and the hazard assessment of AFB_1_ using UV detoxification.

## 1. Introduction

**Figure 1:**
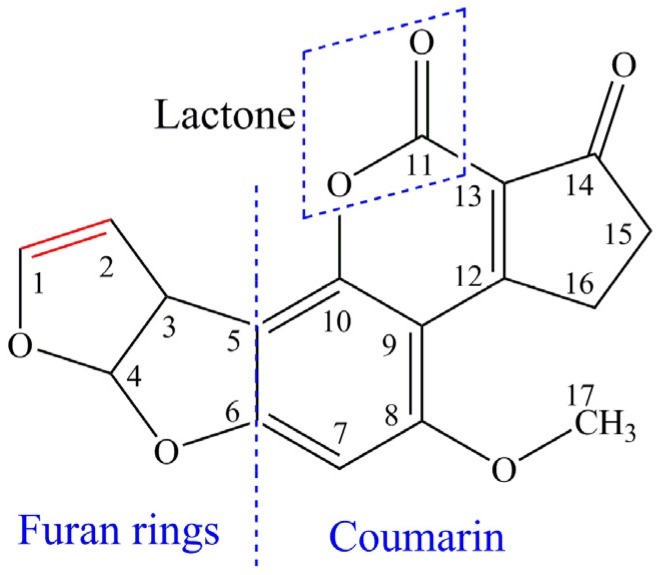
Chemical molecular structure of aflatoxin B_1_ (AFB_1_).

Aflatoxins are the secondary metabolites of *Aspergillus flavus* and *Aspergillus parasiticus*, which are found worldwide in air and soil to infest living and dead plants [1,2]. Aflatoxin B_1_ (AFB_1_) is the most potent mycotoxin associated with hepatocellular carcinoma, immune-dysfunction, and protein deficiency syndromes, which is classified as a Group I carcinogen by the International Agency for Research in Cancer [3]. It is well known that the double bond in the terminal furan ring was a key active site for its toxic and carcinogenic activities. A chemical molecular structure diagram of AFB_1_ is shown in Figure 1. Aflatoxins frequently occur in tropical and subtropical regions, and many agricultural commodities and main crops, such as peanut and corn, are susceptible to such contamination in the period of growth and storage. While the contaminated peanuts or corns are used as raw material without appropriate processing measures in the food industry, they will become a serious threat or hazard to human health. The most rational strategy to avoiding hazards associated with aflatoxins is the prevention of aflatoxin contamination by the toxigenic fungi. However, it is difficult to prevent or control the contamination of peanuts by the toxigenic fungi due to that the hazard of fungi is naturally occurring and is not always possible under certain agronomic storage practices [4]. 

Many kinds of chemical, physical, and biological approaches for the detoxification of aflatoxins have been reported in previous studies [5,6,7,8]. Chemical detoxification is an effective measure using strong alkalis or oxidants such as ammoniation, ozone to break the structure of aflatoxins. However, the quality and nutritional compositions in food may be affected by strong alkalis or oxidants during these chemical measures. Alberts et al. reported that a remarkable reduction in AFB_1_ was observed after 2 h in the presence of *R. erythropolis* extracellular extractions, and only 33.2% residue AFB_1_ was detected after 72 h by degrading enzymes from extracellular extractions [7]. The biological methods showed the high efficiency and selectivity, but these methods may be difficult to reutilize on a large scale. Ultraviolet (UV) irradiation as a non-thermal technology is widely applied in the food industry for disinfection, which is also considered to be practical and cost-effective method to reduce aflatoxins for its photosensitive properties [8]. As an effective physical method, many studies have been done to investigate the efficiency of UV irradiation, the degradation product of aflatoxins, the safety of degradation products and quality of foods after being irradiated [9,10,11,12,13,14]. It was found that aflatoxins could be efficiently degraded by UV irradiation, and the degradation efficiency varied with the differences of irradiation conditions [9,10]. For the studies in photodegradation products of aflatoxins, ultra-performance liquid chromatography-quadrupole time-of-flight mass spectrometry (UPLC-Q-TOF MS) was used to identify the photodegradation products. Different degradation products were identified on the basis of low mass error and high matching property in aqueous or acetonitrile solution, and the different AFB_1_ degradation pathways were proposed [12,14]. Moreover, in other study, it was found that three products of AFB_1_ formed using UV irradiation for 120 min in the presence of methylene blue [15]. These conclusions indicated that depending upon reaction conditions such as medium or solvent, the degradation products of AFB_1_ was different under UV irradiation.

In addition, most studies were carried out on the Ames test and cytotoxicity of HepG2 cells of AFB_1_ products after irradiation [10,13,16,17]. The toxicities of the photodegradation products of AFB_1_ in water and peanut oil on HepG2 cells were investigated, and it was found that the cytotoxicity of products of AFB_1_ in water decreased 40%, while that of products of AFB_1_ in peanut oil reduced about 100% [13]. Moreover, similar results were obtained after being irradiated of AFB_1_ in peanut oil using a photodegradation reactor in a previous study [16]. This might be because toxicological sites were destroyed by UV irradiation.

From the above studies, it can be determined that the studies on degradation products of AFB_1_ are mostly in acetonitrile or aqueous media, and the pathway of AFB_1_ in different solutions are entirely different. However, the identification of degradation products and their toxicity have been poorly investigated in practical production. For instance, the product of AFB_1_ in peanut oil under UV irradiation has not yet been reported, which may be due to the complicated compositions in peanut oil. Therefore, the aim of this article is to examine the products of AFB_1_ in peanut oil under UV irradiation and the safety or toxicity of degradation products after UV irradiation, and to provide clues to the study of the degradation mechanism of AFB_1_ in peanut oil and the assessment of safety issues of the UV method applied in aflatoxins detoxification. In this study, the photodegradation efficiency of AFB_1_ in peanut oil were investigated; after optimizing the extract conditions, the photodegradation products were analyzed by Thermo Quadrupole Exactive Focus spectrometry-mass spectrometry/mass spectrometry (TQEF-MS/MS). On the basis of low mass error and high matching property from data of MS/MS, the feasible pathway of AFB_1_ in peanut oil was deduced. Moreover, the in vitro toxicity of AFB_1_ and its degradation products towards human embryo hepatocytes (L-02 cell) were investigated.

## 2. Results and Discussions

### 2.1. Effect of the AFB_1_ Initial Concentration on Degradation Performance in Peanut Oil

**Figure 2:**
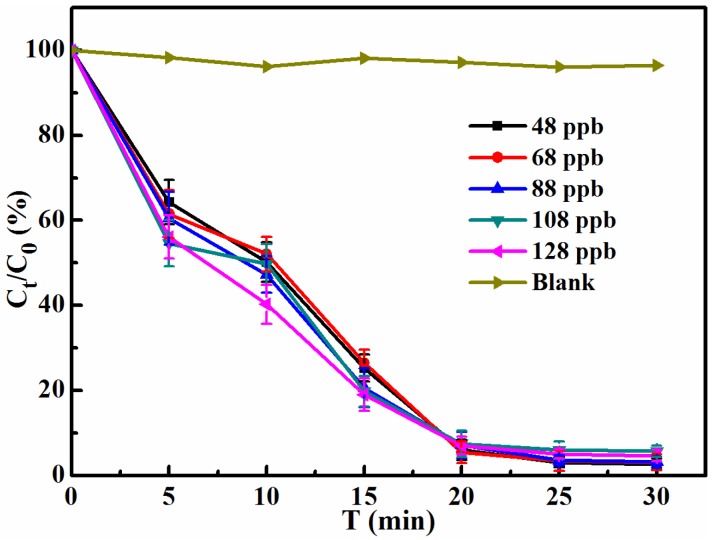
The effect of the AFB_1_ initial concentration on degradation performance in peanut oil under UV irradiation from 0 min to 30 min.

The effect of the AFB_1_ initial concentration on degradation performance in peanut oil was investigated in this study. The result from Figure 2 confirmed that the AFB_1_ can be degraded under 365 nm UV irradiation, and there were no obvious changes found in the blank experiment without UV irradiation. The initial concentrations of AFB_1_ were 48, 68, 88, 108, and 128 ppb, and it could be seen that the initial concentration of 128 ppb showed only a slightly improved degradation efficiency compared with the others before 20 min, and the value of C_t_/C_0_ then remained around 4% after 20 min. It was revealed that the effect of the initial concentration of AFB_1_ in our selected range on degradation activity was unremarkable, which is in agreement with the result of the previous report [12]. Moreover, after 30 min of irradiation, it was found that there was only about 4% AFB_1_ residual in each sample. As we know, many countries have regulated the limitation standard of aflatoxins to protect consumers’ health and prevent food safety issues. The Food and Drug Administration (FDA), the World Health Organization (WHO), and the Codex Alimentarius Commission (CAC) have regulated a maximum contamination level for total content of aflatoxin B1, B2, G1, and G2 (15 μg/kg) in food. Moreover, according to national standard (GB 2461-2011) in China, the limitation standard of AFB_1_ in peanut oil is 20 μg/kg (ppb). It can be concluded that the contents of AFB_1_ in peanut oil with different initial concentrations after 30 min UV treatment in this study are within an acceptable range according to the above limitation standards. However, the European Commission has regulated a maximum contamination level for AFB_1_ (2 μg/kg) and total aflatoxin (4 μg/kg) in argo-products according to (EU) No 165/2010, 2010. For this strict restriction in EU, the optimization of the conditions of UV irradiation treatment, such as enhancing the intensity of UV within a defined and accepted range [12,14], may be an effective strategy to reduce and control the content of AFB_1_ below the limitation standard of EU.

### 2.2. Identification of Photodegradation Products of AFB_1_ in Peanut Oil

**Figure 3:**
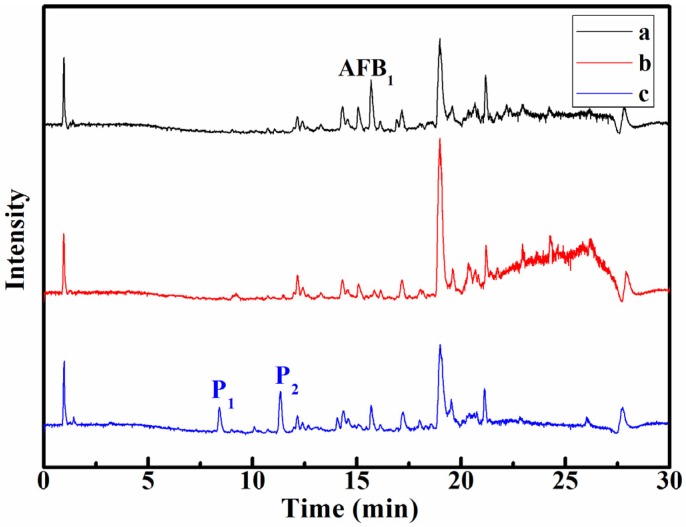
The UPLC chromatogram of peanut oil samples before and after irradiation. ((**a**) the sample containing AFB_1_ without UV irradiation, (**b**) the sample without AFB_1_ after UV irradiation, and (**c**) the sample containing AFB_1_ after UV irradiation).

It is well known that the degradation products of AFB_1_ under UV irradiation in various media are different. Three products, C_17_H_14_O_7_, C_16_H_14_O_6_, and C_16_H_12_O_7_, were formed in aqueous medium and identified by UPLC-Q-TOF-MS [12], while C_17_H_15_O_6_, C_16_H_13_O_5_, and C_14_H_11_O_6_ were obtained in acetonitrile medium by the same identification method [14]. Moreover, in other previous articles, it was reported that the photodegradation products in peanut oil extracted by acetonitrile cannot be detected in UV-treated extracts [9]. In our study, the extraction solvent type and volume were optimized. Various organic reagents were used to extract the photodegradation products of AFB_1_, and it was found that the products extracted by methyl alcohol-water (10:90) solution could be detected. To study the photodegradation products of AFB_1_ and identify their structures, the samples ((a) the sample containing AFB_1_ without UV irradiation; (b) the sample without AFB_1_ after UV irradiation; and (c) the sample containing AFB_1_ after UV irradiation) were analyzed by UPLC-QEF-MS/MS. It can be observed distinctly from Figure 3 that AFB_1_ was degraded within 30 min, leading to the formation of the two new compounds denoted ‘P_1_’ and ‘P_2_’, which were not detected in the blank experiment a and b. Moreover, these two compounds were considered as the irradiated products of AFB_1_ in peanut oil. P_1_, P_2_, and AFB_1_ had a retention time of 8.42, 11.34, and 15.68 min, respectively, showing an order of polar character P_1_ > P_2_ > AFB_1_.

**Figure 4:**
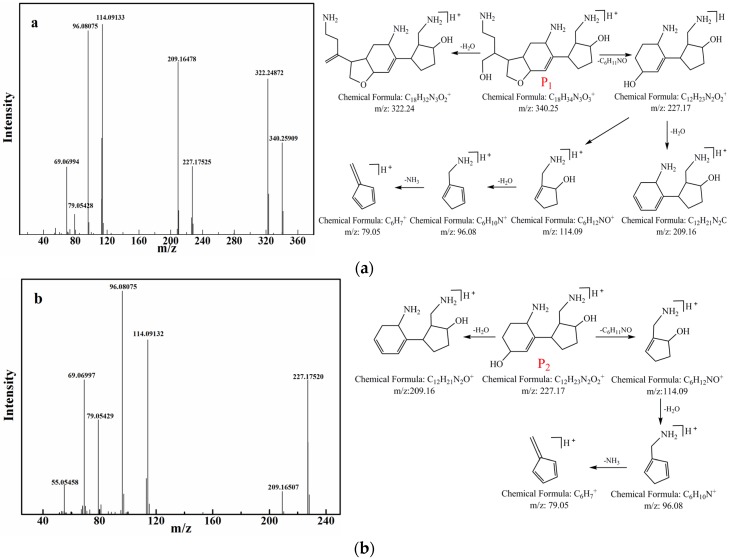
The QEF MS/MS spectra and proposed fragmentation of two degradation products under UV irradiation. Proposed fragmentations are shown on the right side of spectra. (**a**) *m*/*z* = 340; (**b**) *m*/*z* = 227.

**Figure 5:**
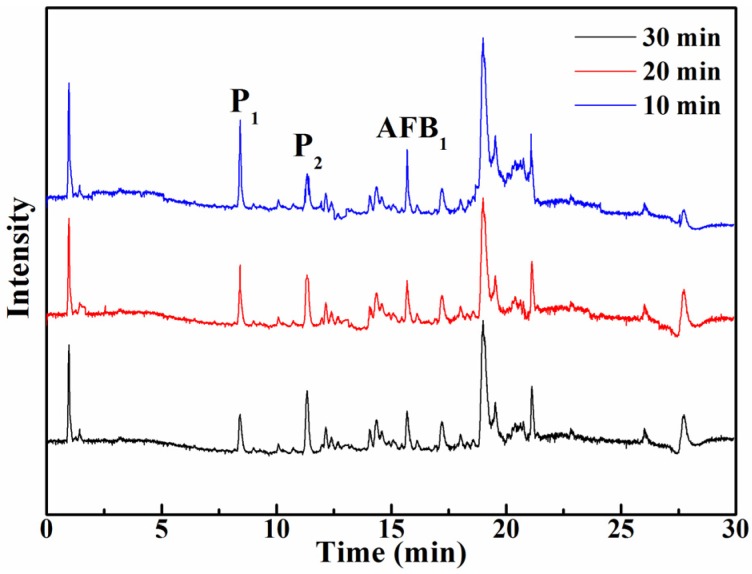
Time development of the formation of photodegradation products of AFB_1_ in peanut oil.

In a separate QEF MS mode, the ions of P_1_, P_2_, and AFB_1_ were used as precursor compounds, and the ion-filtering function of the quadrupole permitted only ions of *m*/*z* = 340, 227, and 313 to pass to the collision cell, where 35 eV was applied for 340, 227, and 313. Compared with the standard substance of AFB_1_, *m*/*z* = 313 was in agreement with the molecular mass of the parent compound (AFB_1_). In order to present the structure of these two products, P_1_ and P_2_, the MS-MS spectra of degradation products *m*/*z* = 340 and *m*/*z* = 227 were also recorded. The secondary mass spectrum and fragmentation information of these two precursor compounds were derived, and they are shown in Figure 4. The structural formulas of these photodegradation products were demonstrated on account of the molecular formula calculated from accurate mass analysis. The MS-MS fragmentation formation was supplied by the QEF mode, and illuminated by the tool mass fragment incorporated in the Mass Frontier 7.0 software to verify how much the results matched the corresponding fragment information (Table 1 contains detailed calculation data). From Table 1, it can be seen that the experimental mass of these fragments matched the theoretical mass of these fragments. Moreover, a maximum mass error of 10 ppm for the range of masses was discussed in this study. It can be seen clearly that the mass errors of these fragments of photodegradation products were all below 7 ppm, which indicated at least 93% confidence in the accuracy of the suggested results. From Figure 4a,b, it can be easily found that *m*/*z* = 340 and *m*/*z* = 227 have many of the same fragmentations such as *m*/*z* = 209, *m*/*z* = 114, *m*/*z* = 96, *m*/*z* = 79, etc. Moreover, through accurate calculation and careful molecular model derivation, we finally arrived at the conclusion that the fragmentation of *m*/*z* = 227 may be the metabolite of *m*/*z* = 340 under UV irradiation. More important than the above results, and to confirm the relationship between these two products, the time development of the formation of photodegradation products of AFB_1_ in peanut oil was also investigated in our study. From Figure 5, it can be seen that P_1_ and P_2_ were detected during the procedure of UV irradiation, and the content of AFB_1_ in peanut oil decreased gradually with time. Moreover, by comparing the ratio of the intensities of two photodegradation products at different time under UV irradiation, it was found that the response and intensity of P_1_ weakened from 10 min to 30 min, while that of P_2_ increased over time. This result may be due to the fact that some of the P_1_ translated to P_2_ during the UV irradiation in peanut oil. These results provided important information for the photodegradation pathway of AFB_1_ in peanut oil.

### 2.3. Proposed Pathway of AFB_1_ under UV Irradiation in Peanut Oil

**Figure 6:**
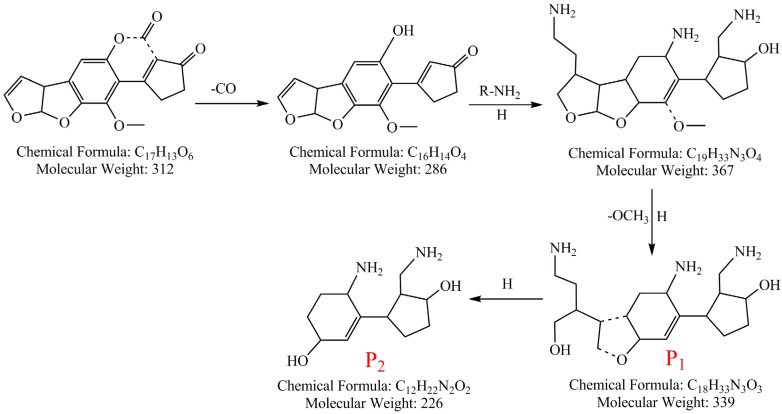
Possible photodegradation pathway of AFB_1_ in peanut oil under 365 nm UV irradiation in this study.

From the above results and discussion, the possible photodegradation pathway of AFB_1_ in peanut oil was proposed, which was based on the identified chemical structure of the photodegradation products and shown in Figure 6. Because there are kinds of compounds such as proteins, amino acids, polyphenols, and other small molecule compounds in peanut oil [18,19], the photodegradation pathways of AFB_1_ in peanut oil might be complicated and accompanied by some complex chemical reactions. As shown in Figure 6, it can be deduced that AFB_1_ might lose the C=O of the lactone ring and become to C_16_H_14_O_4_ firstly, because the lactone ring was the active site of AFB_1_ [20]. Then, the additional reaction and substitution reaction of small molecules such as R-NH_2_ and -NH_2_, which may due to the fact that the concomitant cracking reactions of the nitrogen-containing compound in the peanut oil under UV irradiation occurred on the unsatisfied chemical double bond on the furan ring and the right side five-membered ring of C_16_H_14_O_4_, while the OH groups had been replaced by NH_2_. Moreover, the H addition reaction definitely occurred on C=O [21]. After these complex reactions, the structure of C_19_H_33_N_3_O_4_ was formed. Thus, the P_1_ (C_18_H_33_N_3_O_3_) may be the metabolites of C_19_H_33_N_3_O_4_ after dropping the methoxy group (OCH_3_) under UV irradiation. Finally, as a result of the cracking of the five-membered ring in the middle of the compound of P_1_, the compound of P_2_ (C_12_H_22_N_2_O_2_, molecular weight: 226) formed. Although the proposed photodegradation pathway of AFB_1_ in peanut oil was on the base of photochemical principles and identified structural formulas, the detailed and comprehensive degradation pathway need to be proved further in a future study.

### 2.4. Cell Viability Assay of AFB_1_ and Its Photodegradation Products

**Figure 7:**
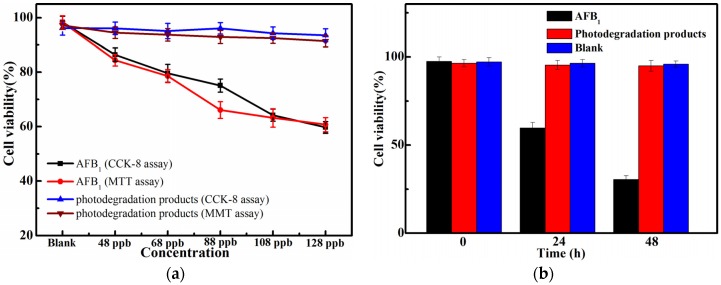
Cell viability assay of AFB_1_ and its photodegradation products. (**a**) The effect of different concentrations of AFB_1_ and the same initial concentrations of its photodegradation products in peanut oil on L-02 liver cells viability evaluated by the MTT and CCK-8 assay. (**b**) L-02 liver cells viability exposed to 128 ppb AFB_1_ and the same concentration photodegradation products for 0, 24, and 48 h evaluated by the CCK-8 assay.

In order to investigate the toxicity and hazard of photodegradation of AFB_1_ after UV irradiation, the viabilities of human embryo hepatocytes (L-02 liver cells) were assessed using the MTT assay and the CCK-8 assay. As shown in Figure 7a, it can be seen that the consequences of the MTT and CCK-8 assays were similar, and the viability of L-02 liver cells decreased in a linear relation with increasing concentrations of AFB_1_ from 48 to 128 ppb after incubation for 24 h. The cell viability treated by the concentration of AFB_1_ at 128 ppb decreased more significantly than that of other samples. Compared with a blank control trial, the cell viability of L-02 liver cells was reduced by about 40% after incubation at a concentration of 128 ppb. However, the loss of cell viability of irradiated samples at the same concentration was significantly decreased compared with that of samples without irradiation treatment, which indicated that the toxicity of the degradation products of AFB_1_ in peanut oil lowered after UV irradiation. Moreover, it can be seen from Figure 7b that the mortality rates of L-02 liver cells was time-dependent in the presence of AFB_1_ while the mortality rates of L-02 liver cells had no obvious changes with time in the presence of the photodegradation products of AFB_1_, and the mortality rate of L-02 liver cells in the presence of AFB_1_ was up to 70% for 48 h. This investigation further confirmed that the cytotoxicity of AFB_1_ was significantly reduced after UV irradiation, which may be due to the changes in the molecular structure of AFB_1_. In this present study, the MS/MS date expounded that P_1_ (C_18_H_33_N_3_O_3_) and P_2_ (C_12_H_22_N_2_O_2_) were formed after the removal of the double bond in the terminal furan ring and the modification or splitting decomposition of the lactone ring, which may be the main reason for the decrease in toxicity after UV irradiation. Moreover, it is well known that the structure of AFB_1_ contained a lactone ring and a furan moiety, and the double bond in the terminal furan ring was the key active site for its toxic and carcinogenic activities [22,23,24]. This viewpoint is also confirmed by a previous study that the toxicity of radiolytic products was significantly reduced compared with that of AFB_1_ because of the addition reaction that occurred on the double bond in the terminal furan ring [25]. Lee et al. found that the lactone ring played a significant role in the fluorescence of AFB_1_, and the AFB_1_ without the lactone ring became non-florescent with a subsequent decline in toxicity [26]. Therefore, it could be deduced that, due to the reaction on the double bond of the terminal furan ring and the lactone ring for AFB_1_ in this study, the toxicity of the two degradation products decreased after UV irradiation treatment compared with that of AFB_1_.

## 3. Conclusions

To our knowledge, this is the first report to demonstrate the proposed degradation products and pathway of AFB_1_ in peanuts oil under UV irradiation. The products of AFB_1_ in peanut oil under UV irradiation were extracted by methyl alcohol and deionized water and analyzed by UPLC-TQEF-MS/MS. It was found that two photodegradation products (P_1_:C_18_H_33_N_3_O_3_ and P_2_:C_12_H_22_N_2_O_2_) were formed. Moreover, reactions of photodegradation mainly occurred on the terminal furan ring and the lactone ring, which were the primary toxicological sites of AFB_1_. After the resolution of accurate mass spectra and a comparison of UPLC spectra at different times during irradiation, the metabolic pathways of AFB_1_ in peanut oil were proposed. In addition, the toxicity assessment of degradation products through a human embryo hepatocytes viability assay revealed that the toxicity of degradation products decreased significantly compared with that of AFB_1_, which reaffirmed that the reactions occurred on the primary toxicological sites of AFB_1_ under irradiation. The present findings could provide a new idea and means for future studies on metabolic pathways and toxicity assessments of hazardous substances in foodstuffs.

## 4. Materials and Methods

### 4.1. Materials and Synthesis

AFB_1_ (2, 3, 6α, 9α-tetrahydro-4-methoxycyclopenta [c] furo [2, 3:45] furo [2, 3-h] chromene-1, 11-dione; purity >98%) was obtained from Sigma (Sigma-Aldrich, St. Louis, MO, USA). Methyl alcohol, dimethyl sulfoxide (DMSO), and other reagents are analytical reagents and purchased from Sinopharm (Sinopharm Chemical Reagent Co., Ltd., Shanghai, China) Peanut oil was purchased from Wal-Mart supermarket in Wuhan, China.

For high-performance liquid chromatography (HPLC) and TQEF-MS/MS analysis, chromatographic grade acetonitrile and methyl alcohol were purchased from Sigma (St. Louis, MO, USA). The deionized water was obtained from a Milli-Q SP Reagent Water system (Millipore, Bedford, MA, USA).

For cytotoxicity tests, human embryo hepatocytes (L-02 cells) were purchased from Procell (Procell Co. Ltd., Wuhan, China). Culture medium RPMI-1640 and fetal calf serum were purchased from HyClone ((GE-Healthcare, Logan, UT, USA). The 0.05% trypsin solution was from Thermo Fisher (Thermo Fisher Scientific, Waltham, MA, USA).

### 4.2. Photodegradation Treatment

For UV degradation experiments, 20 g of peanut oil with different initial concentrations of 48 ppb, 68 ppb, 88 ppb, 108 ppb, and 128 ppb AFB_1_ were put in glass petri dishes and irradiated under a 100 W ultraviolet lamp (λ = 365 nm, 55–60 mw/cm^2^, Changzhou YuYu Electro-Optical Device Co., Ltd., Changzhou, China). The photoreaction was performed in a close reaction box in a temperature-controlled room, and temperature was kept at 26 °C. The distance between the UV lamp and the peanut oil was 40 cm. At certain selected time intervals, irradiated samples were collected and analyzed via HPLC (Agilent 1100, Agilent Technologies, Santa Clara, CA, USA). Each test was repeated five times.

#### 4.2.1. Extraction and Instrumental Analysis for AFB_1_ in Peanut Oil

A total of 2.5 g of peanut oil was weighed after UV treatment in a 50 mL centrifuge tube, and 7.5 mL of acetonitrile was then added. The mixture was shaken for 5 min with a platform shaker at room temperature and extracted by sonicator (Nanjing Jiancheng Bioengineering Institute, Nanjing, China) for 10 min. Then, the mixture was centrifuged at 7000 rpm for 15 min, and the precipitate was discarded. A 1.5 mL supernatant with 4 mL of deionized water was collected in a clean test tube. Then, the above mixture was filtered through a 0.22 µm organic ultra-filter membrane (Agilent Technologies, Santa Clara, CA, USA). The filtrate was passed through immunoaffinity columns and washed with 1 mL of methyl alcohol into a glass tube.

HPLC-FLD analysis was performed on an Agilent 1100 system equipped with a fluorescence detector, an auto-injector, and a quaternary solvent-delivery system. The chromatographic column was a 150 mm × 4.6 mm Agilent C_18_ column (Agilent Technologies, Santa Clara, CA, USA) with a 5 µm particle size. The injection volume was 10 µL. The mobile phases were methyl alcohol (A) and aqueous solution (B) in a 45:55 (*V_A_*/*V_B_*) solution with a flow rate of 0.7 mL∙min^−1^. The detection of the excitation and emission wavelengths were at 360 nm and 440 nm, respectively

#### 4.2.2. Test Compounds of Photodegradation Products

The mixtures of photodegradation products in peanut oil was extracted by a methyl alcohol-water solution with a volume ratio of 10:90, and then passed through a 0.22 µm organic ultrafilter membrane (Agilent Technologies, Santa Clara, CA, USA) into a glass tube.

The samples were analyzed and recorded by Ultra Performance Liquid Chromatograph-Thermo Quadrupole Exactive Focus mass spectrometry/mass spectrometry (UPLC-TQEF-MS/MS, Thermo Fisher Scientific, Waltham, MA, USA). UPLC was performed on a Thermo system with a quaternary solvent-delivery system and an autosampler. The injection volume was 10 µL. The chromatographic column was presented on a 100 mm × 2.1 mm Thermo C_18_ column (Thermo Fisher Scientific, Waltham, MA, USA) with a particle size of 5 µm. The mobile phase was a gradient prepared from acetonitrile (A) and a 0.2% formic acid aqueous solution (B). The elution began with 5% A for 0.1 min, and the proportion of A was increased linearly to 100% at 36 min, and then brought back to 5% A at 40 min and kept for 5 min for a total of 45 min. The flow rate of the mobile phase was kept at 200 µL∙min^−1^.

Mass spectrometry was performed on a Thermo Quadrupole Exactive Focus (QEF) system (Thermo Fisher Scientific, Waltham, MA, USA). The products were analyzed under positive-ion (PI) mode. The optimized conditions were Sheath gas 35 L∙min^−1^, Aux gas (Ar) 5 L∙min^−1^, and capillary potentials at 3000 kV. The QEF instrument was operated in full scan mode, and data were collected between *m*/*z* = 50 and 500, with a scan accumulation time of 0.2 s. The MS/MS experiments were performed using a collision energy of 35 eV, which was optimized for each product.

### 4.3. Cell Viability Assay

#### 4.3.1. Cell Culture

L-02 liver cells were serially cultivated in the RPMI-1640 complete medium (15% *v*/*v* fetal calf serum, 100 U/mL penicillin, 100 U/mL streptomycin) at 37 °C, with 4% CO_2_ in air until an 80% degree of fusion was reached, and then incubated with DMSO, in which the final concentration of DMSO was less than 0.1%.

#### 4.3.2. Cell Viability Assay

A total of 1 × 10^3^ cells were seeded in a 96-well plate, allowing the attachment of cells to the substrate for 6–8 h, and the cells were then divided into different groups (six wells per group). For the sample of photodegradation products, the methyl alcohol-water solution with a volume ratio of 10:90 was used to extract the products of AFB_1_ in peanut oil after UV irradiation, and the products were then evaporated under a stream of nitrogen and then collected in DMSO for cell viability assay. Cells were exposed to different concentrations of AFB_1_, photodegradation products of AFB_1_ and DMSO were used as a control, and cultivation continued for 24 h and 48 h. Cell viability was determined with a MTT assay and a CCK-8 assay. In brief, for the MTT method, 20 µL of 5 mg∙mL^−1^ MTT in PBS buffer solution was added to each well, and the plate was incubated at 37 °C for 4 h. After incubation, the medium was then discarded, and the formed crystals were dissolved in 150 µL of DMSO. The absorbance of each well was then measured at 490 nm using a microplate reader (SpectraMax M2e, Molecular Devices, Sunnyvale, CA, USA), and the percentage viability was calculated. Moreover, for the CCK-8 assay, 10 µL of the CCK-8 solution was added to each well, and the plate was incubated at 37 °C for 4 h. After incubation, the absorbance of each well was measured at 450 nm using a microplate reader (SpectraMax M2e, Molecular Devices, Sunnyvale, CA, USA), and the percentage viability was calculated.

## Figures and Tables

**Table 1:** QEF-MS/MS accurate mass, mass error, and formula of photodegradation product fragments.

Theoretical Mass	Experimental Mass	Fragment Formula	Mass Error (ppm)
79.05478	79.05429	C_6_H_7_	−6.19823
79.05478	79.05428	C_6_H_7_	−6.32473
96.08132	96.08075	C_6_H_10_N	−5.93247
114.09189	114.09132	C_6_H_12_NO	−4.99597
114.09189	114.09133	C_6_H_12_NO	−4.90832
209.16539	209.16478	C_12_H_21_N_2_O	−2.91635
209.16539	209.16507	C_12_H_21_N_2_O	−1.52989
227.17595	227.17520	C_12_H_23_N_2_O_2_	−3.30141
227.17595	227.17525	C_12_H_23_N_2_O_2_	−3.08131
340.26002	340.25909	C_18_H_34_N_3_O_3_	−2.73320
